# Mortality among adults: gender and socioeconomic differences in a Brazilian city

**DOI:** 10.1186/1471-2458-12-39

**Published:** 2012-01-17

**Authors:** Ana Paula Belon, Marilisa BA Barros, Letícia Marín-León

**Affiliations:** 1 Department of Collective Health, School of Medical Sciences, State University of Campinas, São Paulo, Brazil

**Keywords:** Health Inequalities, Social Inequalities, Socioeconomic status, Mortality, Causes of Death, Gender, Brazil

## Abstract

**Background:**

Population groups living in deprived areas are more exposed to several risk factors for diseases and injuries and die prematurely when compared with their better-off counterparts. The strength and patterning of the relationships between socioeconomic status and mortality differ depending on age, gender, and diseases or injuries. The objective of this study was to identify the magnitude of social differences in mortality among adult residents in a city of one million people in Southeastern Brazil in 2004-2008.

**Methods:**

Forty-nine health care unit areas were classified into three homogeneous strata using 2000 Census small-area socioeconomic indicators. Mortality rates by age group, sex, and cause of death were calculated for each socioeconomic stratum. Mortality rate ratios (RR) and 95% confidence intervals were estimated for the low and middle socioeconomic strata compared with the high stratum.

**Results:**

In general, age-specific mortality rates showed a social gradient of increasing risks of death with decreasing socioeconomic status. The highest mortality rate ratios between low and high strata were observed in the 30-39 age group for males (RR = 1.74, 95% CI 1.59-1.89), and females (RR = 1.90, 95% CI 1.65-2.15). Concerning specific diseases and injuries, the greatest inequalities between low and high strata were found for homicides (RR = 2.44, 95% CI 2.27-2.61) and traffic accidents (RR = 1.64, 95% CI 1.45-1.83) among males. For women, the highest inequalities between the low and high strata were for chronic respiratory diseases (RR = 2.19, 95% CI 1.94-2.45) and acute myocardial infarction (RR = 1.93, 95% CI 1.79-2.07). Only breast cancer showed a reversed social gradient (RR = 0.70, 95% CI 0.48-0.92). Inequalities in circulatory and respiratory diseases mortality were greater among females than among males.

**Conclusions:**

Substandard living conditions are related to unhealthy behaviors, as well as difficulties in accessing health care. Therefore, the Brazilian Health System (SUS) must ensure greater access to primary and hospital care, and develop programs that promote healthier lifestyles among vulnerable groups to reduce social inequalities in mortality. Moreover, because deaths from external causes are concentrated in poor areas, cooperative and coordinated intersectoral actions should be taken to combat the deadly violence cycle.

## Background

Population groups with low levels of education and per capita income who live in poor conditions have greater exposure to several risk factors for diseases and injuries, are less likely to visit a doctor, and are more likely to get sick often and die prematurely, compared with their better-off counterparts. This is confirmed by several studies examining the relationship between socioeconomic status and health [1-4]. There is a consensus on the existence of a social gradient in health and mortality regardless of the affiliated theoretical currents, the applied methodology, the individual or aggregated approach, or even the chosen socioeconomic variables.

The strength and patterning of the relationship between socioeconomic status and mortality differ depending on age [2,5], gender [6,7], and diseases or injuries [3,8]. Disagreement can be found in the literature regarding the magnitude or even the direction of association between social status and mortality for some specific diseases. These differences can only be partially explained by study design, variables used in the social stratification, study period, or territorial unit of analysis [9].

At the individual level, researchers have investigated the social inequalities in mortality based primarily on social classes defined by occupation [2,10,11], as well as education and/or income [12-15]. At the aggregated level, by applying spatial approaches, geographic variations in health and mortality have been identified among localities with different socioeconomic characteristics [1,16,17].

In Brazil, the scientific literature on this topic is limited, especially when considering the profound social inequalities plaguing the country [18-21]. Moreover, the decrease in mortality has stimulated interest in measuring mortality indicators within society, to assess how different population segments have benefited from the reduction in the risk of death. Thus, it is imperative to examine the different faces of mortality under unequal living conditions with reference to age, gender, and underlying cause of death.

Therefore, this study aims to identify the magnitude of social inequalities in mortality, among adult residents in a large Brazilian city in the period 2004-2008.

## Methods

A descriptive ecological study of mortality indicators stratified by socioeconomic status was conducted in the city of Campinas in the period 2004-2008. Campinas is an industrial city with a population of over one million inhabitants located in the State of São Paulo, which is the wealthiest state in Brazil.

Social differences in mortality were analysed using an ecological approach. To provide better health assistance to the population, the total area of Campinas was divided by the Municipal Health Department (MHD) into 49 health care unit areas, which are used as the analysis units of this study. The population size of these areas ranges from 2924 to 69 155, with a median of 19 010. Population size, socioeconomic variables, and the number of deaths for the coverage areas of the 49 primary health care units of Campinas were provided by the MHD. Because population and socioeconomic data from the Brazilian 2000 Census were provided by census tract, the MHD identified the census units that correspond to each of the 49 health care unit areas using geocoding techniques.

The socioeconomic strata were determined by the head of each household's educational attainment and income levels. Two education indicators were defined: proportion of heads of household with (1) less than one year and (2) more than 10 years of schooling. The monthly income indicators were: proportion of heads of household (1) earning less than two minimum wages, and (2) earning equal to or more than 10 minimum wages. These four indicators were chosen because they demonstrate a strong discriminatory capacity compared with other indicators. The 49 health care unit areas were ranked according to each indicator. The ranking always ranged from the worst to the best socioeconomic condition, assigning the first position to the most socio-economically vulnerable area. The average position of the four indicators determined the overall score of each area, which in turn established a hierarchical classification according to socioeconomic status. The 49 health care unit areas were divided into low, middle and high socioeconomic strata. Following the ranking, approximately one-third of the total population was represented in each stratum by adding up the population of each area until around 33.3% of Campinas population was obtained.

For the year 2006, the population projections of the 49 health care unit areas were calculated by the MHD using the Aibi method [22]. This method allows projected population calculations of small areas based on the population growth trend of the total area where they are located.

Deaths were categorized by strata based on usual residence in a particular area defined from the address. The analyses focused only on people aged 20 or older, because for the majority of selected specific diseases (with the exception of prostate cancer), deaths only occurred beyond this age. Mortality data were classified by ICD-10 codes (International Classification of Diseases, 10th revision).

All mortality rates for the population aged 20 years or older were calculated by sex and for each socioeconomic stratum, using average deaths over the period 2004-2008 and 2006 population data. Cause-specific and overall mortality rates were age-adjusted by direct method using the 2000 Campinas total population age structure as the standard. Specific rates for 10-year age groups (except for the last open-interval age group) were also estimated.

Mortality rates were estimated for the five main groups of causes of death. The following ICD-10 chapters were analysed for both sexes: malignant neoplasms (chapter 2), circulatory diseases (chapter 9), respiratory diseases (chapter 10), digestive diseases (chapter 11), and external causes (chapter 20). Cause-specific mortality rates were estimated for those underlying causes of death with at least 60 cases in each sex. This cut-off point was used to enable a minimum number of deaths for comparison among strata, preventing random fluctuations in mortality rates caused by small numbers of deaths. Among the male population the following causes of death were included: acute myocardial infarction (I21), cerebrovascular diseases (I60-I69), pneumonia (J12-J18), lower chronic respiratory diseases (J40-J47), traffic accidents (V01-V89), homicide (X85-Y09), stomach cancer (C16), lung cancer (C33-C34) and prostate cancer (C61). Prostate cancer was not observed among men younger than 40 years in our sample, therefore data of this cancer was restricted to men aged 40 years and older. Among females, malignant breast cancer (C50), acute myocardial infarction (I21), cerebrovascular diseases (I60-I69), pneumonia (J12-J18), and lower chronic respiratory diseases (J40-J47) were analysed.

The Armitage method [23] was used to estimate 95% confidence intervals for the mortality rate ratios (RR) of the low and middle socioeconomic strata, using the high stratum rates as reference.

As all data used in the present study are openly available from government websites, ethical clearance was not necessary.

## Results

Population number, households, and head of household indicators showed large socioeconomic differences among strata (Table 1). The low socioeconomic stratum was characterized by a high proportion of young people under 15 years (31.1%) and a small proportion of elderly people (4.9%). Therefore, the aging index (elder-child ratio) was only 15.9%, meaning that there were only 15.9 people aged 60 or older for every 100 individuals aged less than 15 years old. The aging index in the high stratum was five times higher than in the low stratum. About 25% of the households in the low socioeconomic stratum were located in slum areas and only 0.2% had four or more bathrooms. In this stratum, only 4.4% of the heads of households earned 10 or more monthly minimum wages and 2.4% completed higher education.

**Table 1:** Demographic and social indicators by socioeconomic strata. Campinas, 2000.

Indicators	Socioeconomic strata			Campinas
		
	Low	Middle	High	
Population aged 0 to 14 (%)	31.1	22.6	17.9	24.0

Population aged 60 or over (%)	4.9	9.9	14.0	9.5

Aging Index^1^	15.9	43.7	78.5	39.8

Total Dependency Ratio (%)	56.4	48.2	46.9	50.5

Households in slum areas (%)	24.9	9.1	1.3	11.1

Households with bathrooms or toilets connected to sewerage system (%)	72.3	89.6	93.4	85.6

Households with four or more bathrooms (%)	0.2	2.8	8.1	3.9

Heads of household with an average monthly income of less than 2 minimum wages (%)	28.6	18.4	9.9	18.1

Heads of household with an average monthly income equal to or more than 10 minimum wages (%)	4.4	21.0	44.5	25.2

Heads of household with less than 1 year of schooling (%)	9.8	5.9	2.5	5.9

Heads of household with more than 10 years of schooling (%)	13.7	33.8	60.4	37.3

Heads of household with complete Elementary Education (8 years of schooling) (%)	40.8	21.6	11.3	23.8

Heads of household with complete Higher Education (%)	2.4	14.6	36.3	18.7

**Total Population in 2000 ^2^**	**329 567**	**324 797**	**313 793**	**968 157**

**Total Population in 2006 ^2^**	**396 227**	**334 057**	**309 113**	**1 039 397**

**Source**: Based on data from the 2000 Demographic Census/Brazilian Institute of Geography and Statistics (IBGE).

^1 ^Aging index was estimated using the population aged 60 or over.

^2 ^Population data were provided by the Municipal Health Department (MHD).

In general, the highest mortality rates occurred in the low stratum (Table 2). The overall age-standardized mortality rates for both sexes in the low and middle socioeconomic strata were different to rates in the high stratum. In comparison with affluent areas, overall age-standardized mortality rates for males and for females were respectively 1.17 (95% CI 1.13-1.20) and 1.30 (95% CI 1.25-1.34) higher in the lowest stratum. For all age-specific mortality rates, there was a generally consistent gradient of increasing mortality with decreasing socioeconomic status. The largest mortality rate ratios between the low and high socioeconomic strata were observed among males aged 20-29 (RR = 1.60, 95% CI 1.44-1.76) and 30-39 (RR = 1.74, 95% CI 1.59-1.89). In the female population, only the oldest age group (RR = 1.04, 95% CI 0.96-1.12) showed no differences between low and high strata. Between the low and high strata, the highest inequality was found among females aged 30-39, with a mortality rate ratio of 1.90 (95% CI 1.65-2.15).

**Table 2:** Age-standardized mortality rates^1 ^and age-specific mortality rates (per 1000) by socioeconomic strata, for the male and female population aged 20 and older. Rate ratios in the low and middle socioeconomic strata use high stratum rates as reference. Campinas, 2004-2008.

Mortality rates	Men					Women				
	
	Low^2^	Middle^2^	High^2^	Rate Ratios (95% CI)^3^		Low^2^	Middle^2^	High^2^	Rate Ratios (95% CI)^3^	
	
	a	b	c	a/c	b/c	d	e	f	d/f	e/f
**Overall mortality rates (20 years or more)**	**11.09**	**11.18**	**9.50**	**1.17**	**1.18**	**7.11**	**6.50**	**5.48**	**1.30**	**1.19**
	
	(867)	(1060)	(1124)	(1.13-1.20)	(1.14-1.21)	(546)	(801)	(1044)	(1.25-1.34)	(1.14-1.23)

**Age-specific**										

**20 to 29**	2.16	1.58	1.35	**1.60**	1.17	0.42	0.42	0.26	**1.64**	**1.64**

	(83)	(50)	(37)	(1.44-1.76)	(0.99-1.34)	(17)	(13)	(7)	(1.28-2.01)	(1.27-2.00)

**30 to 39**	2.94	2.28	1.69	**1.74**	**1.35**	1.10	0.89	0.58	**1.90**	**1.53**

	(95)	(62)	(40)	(1.59-1.89)	(1.19-1.51)	(37)	(25)	(15)	(1.65-2.15)	(1.27-1.79)

**40 to 49**	5.41	5.20	3.73	**1.45**	**1.39**	2.49	1.87	1.94	**1.29**	0.97

	(124)	(116)	(79)	(1.33-1.57)	(1.28-1.51)	(58)	(46)	(48)	(1.12-1.45)	(0.79-1.14)

**50 to 59**	14.07	10.45	9.80	**1.44**	1.07	7.44	5.19	4.59	**1.62**	1.13

	(169)	(151)	(144)	(1.35-1.53)	(0.97-1.16)	(87)	(84)	(78)	(1.49-1.75)	(0.99-1.27)

**60 to 69**	26.13	25.05	19.00	**1.37**	**1.32**	14.96	13.05	9.81	**1.52**	**1.33**

	(150)	(224)	(189)	(1.29-1.46)	(1.23-1.40)	(95)	(136)	(122)	(1.41-1.64)	(1.21-1.44)

**70 to 79**	60.48	76.61	52.06	**1.16**	**1.47**	43.03	37.21	28.21	**1.53**	**1.32**

	(146)	(260)	(303)	(1.09-1.23)	(1.40-1.54)	(124)	(209)	(250)	(1.44-1.61)	(1.23-1.41)

**80 or older**	132.02	163.49	159.48	**0.83**	1.03	131.49	137.23	126.39	1.04	**1.09**

	(100)	(197)	(332)	(0.75-0.90)	(0.95-1.10)	(128)	(288)	(524)	(0.96-1.12)	(1.01-1.16)

^1 ^Age adjusted mortality rates were estimated by the direct method, using the 2000 Campinas total population age structure as the standard.

**^2 ^**The number of deaths in each socioeconomic stratum is indicated in parenthesis.

^3 ^Significant differences at the 5% level are indicated in bold type.

Table 3 presents mortality rates and rate ratios for groups of causes of death among socioeconomic strata. Most groups of causes of death showed consistent trends of decreasing mortality with increasing socioeconomic status. There were differences in mortality rates between the strata for all causes of death in both sexes. Among men, the highest social inequality in mortality between the low and high socioeconomic strata was observed for external causes (RR = 1.99, 95% CI 1.89-2.10). Between the middle and high strata, the greatest rate ratio was for respiratory diseases (RR = 1.44, 95% CI 1.35-1.53). Among women, rate ratios between the low and high socioeconomic strata were remarkably high for respiratory diseases (RR = 1.82, 95% CI 1.70-1.94) and cardiovascular diseases (RR = 1.71, 95% CI 1.64-1.79). In the middle stratum, cardiovascular diseases showed the greatest rate ratio with 1.48 (95% CI 1.40-1.56) higher mortality than in the highest socioeconomic stratum.

**Table 3:** Age-standardized mortality rates^1 ^(per 100 000) by causes of death and socioeconomic strata, for the male and female population aged 20 years and older. Rate ratios in the low and middle socioeconomic strata use high stratum rates as reference. Campinas, 2004-2008.

Groups of causes of death	Men					Women				
	
	Low^2^	Middle^2^	High^2^	Rate Ratios (95% CI)^3^		Low^2^	Middle^2^	High^2^	Rate Ratios (95% CI)^3^	
	
	**a**	**b**	**c**	**a/c**	**b/c**	**d**	**e**	**f**	**d/f**	**e/f**

**Malignant neoplasms**	209.35	213.69	176.68	**1.18**	**1.21**	133.71	133.14	115.15	**1.16**	**1.16**
	
	(145)	(205)	(212)	(1.11-1.26)	(1.13-1.29)	(107)	(165)	(196)	(1.06-1.26)	(1.06-1.25)

**Circulatory diseases**	375.52	380.50	276.65	**1.36**	**1.38**	277.97	240.53	162.22	**1.71**	**1.48**
	
	(257)	(353)	(334)	(1.30-1.42)	(1.31-1.44)	(199)	(297)	(319)	(1.64-1.79)	(1.40-1.56)

**Respiratory diseases**	157.45	168.69	116.98	**1.35**	**1.44**	113.80	86.04	62.61	**1.82**	**1.37**
	
	(99)	(146)	(142)	(1.25-1.44)	(1.35-1.53)	(76)	(106)	(132)	(1.70-1.94)	(1.25-1.50)

**Digestive diseases**	75.99	71.44	55.57	**1.37**	**1.29**	37.1	33.0	25.3	**1.47**	**1.31**
	
	(62)	(72)	(65)	(1.23-1.50)	(1.15-1.42)	(28)	(41)	(46)	(1.27-1.67)	(1.10-1.51)

**External causes**	161.61	108.95	81.16	**1.99**	**1.34**	25.8	26.6	20.5	**1.26**	**1.30**
	
	(181)	(117)	(86)	(1.89-2.10)	(1.23-1.46)	(25)	(32)	(35)	(1.03-1.49)	(1.07-1.52)

^1 ^Age adjusted mortality rates were estimated by the direct method, using the 2000 Campinas total population age structure as the standard.

^2 ^The number of deaths in each socioeconomic stratum is indicated in parenthesis.

^3 ^Significant differences at the 5% level are indicated in bold type.

In relation to cause-specific mortality among men (Table 4), only lung cancer showed no differences between low and high strata (RR = 1.01, 95% CI 0.82-1.20). However, homicides (RR = 2.44, 95% CI 2.27-2.61) and traffic accidents (RR = 1.64, 95% CI 1.45-1.83) showed the largest differences between these strata. Homicides (RR = 1.63, 95% CI 1.45-1.81) and chronic lower respiratory diseases (RR = 1.55, 95% CI 1.38-1.71) demonstrated the greatest rate ratios between middle and high strata.

**Table 4:** Age-standardized mortality rates^1 ^(per 100 000) by causes of death and socioeconomic strata, for the male population aged 20 years and older. Rate ratios in the low and middle socioeconomic strata use high stratum rates as reference. Campinas, 2004-2008.

Specific diseases and injuries	Low^2^	Middle^2^	High^2^	Rate Ratios (95% CI)^3^	
	
	a	b	c	a/c	b/c
**Malignant neoplasms**					

Stomach	49.27	42.25	36.91	**1.33**	1.14
	
	(33)	(42)	(44)	(1.17-1.50)	(0.97-1.32)

Lung	33.67	32.74	33.41	1.01	0.98
	
	(23)	(32)	(41)	(0.82-1.20)	(0.79-1.17)

Prostate^4^	65.45	48.09	47.42	**1.38**	1.01
	
	(16)	(19)	(28)	(1.17-1.59)	(0.78-1.24)

**Circulatory diseases**					

Acute myocardial infarction	148.59	133.94	101.06	**1.47**	**1.33**
	
	(109)	(130)	(121)	(1.37-1.57)	(1.22-1.43)

Cerebrovascular diseases	105.55	95.05	70.72	**1.49**	**1.34**
	
	(70)	(87)	(86)	(1.37-1.61)	(1.22-1.47)

**Respiratory diseases**					

Pneumonia	83.75	84.57	60.11	**1.39**	**1.41**
	
	(55)	(73)	(72)	(1.26-1.52)	(1.28-1.54)

Chronic lower respiratory diseases	48.62	57.23	36.98	**1.31**	**1.55**
	
	(27)	(49)	(46)	(1.15-1.48)	(1.38-1.71)

**External causes**					

Traffic accidents	44.02	28.78	26.78	**1.64**	1.07
	
	(50)	(31)	(28)	(1.45-1.83)	(0.87-1.28)

Homicides	70.12	46.88	28.76	**2.44**	**1.63**
	
	(88)	(52)	(29)	(2.27-2.61)	(1.45-1.81)

^1 ^Age adjusted mortality rates were estimated by the direct method, using the 2000 Campinas total population age structure as the standard.

^2 ^The number of deaths in each socioeconomic stratum is indicated in parenthesis.

^3 ^Significant differences at the 5% level are indicated in bold type.

^4 ^The mortality rates from prostate cancer are calculated for the male population aged 40 and older.

For females, the five diseases examined presented significant social inequality in mortality (Table 5). Between the low and high strata, the highest rate ratios were found for lower chronic respiratory diseases (RR = 2.19, 95% CI 1.94-2.45) and acute myocardial infarction (RR = 1.93, 95% CI 1.79-2.07). In the middle stratum, rate ratios were the highest for chronic respiratory diseases (RR = 1.71, 95% CI 1.45-1.98) and acute myocardial infarction (RR = 1.58, 95% CI 1.44-1.73), compared with the high stratum. In contrast with the other analysed causes of death, breast cancer mortality rates were higher in the high stratum than in the low stratum, presenting a reversed social gradient. In comparison with the high stratum, the low stratum presented a rate ratio of 0.70 (95% CI 0.48-0.92), which means that the breast cancer mortality rate was nearly 45% greater among women in the high stratum.

**Table 5:** Age-standardized mortality rates^1 ^(per 100 000) by causes of death and socioeconomic strata, for the female population aged 20 years or older. Rate ratios in the low and middle socioeconomic strata use high stratum rates as reference. Campinas, 2004-2008.

Specific diseases	Low^2^	Middle^2^	High^2^	Rate Ratios (95% CI)^3^	
	
	a	b	c	a/c	b/c
**Malignant neoplasms**					

Breast	20.47	22.58	29.19	**0.70**	**0.77**
	
	(19)	(28)	(47)	(0.48-0.92)	(0.56-0.99)

**Circulatory diseases**					

Acute myocardial infarction	88.27	72.40	45.79	**1.93**	**1.58**
	
	(65)	(90)	(84)	(1.79-2.07)	(1.44-1.73)

Cerebrovascular diseases	85.87	69.02	47.48	**1.81**	**1.45**
	
	(61)	(85)	(93)	(1.67-1.95)	(1.31-1.60)

**Respiratory diseases**					

Pneumonia	67.45	49.57	36.73	**1.84**	**1.35**
	
	(44)	(61)	(80)	(1.68-1.99)	(1.18-1.52)

Chronic lower respiratory diseases	29.05	22.69	13.24	**2.19**	**1.71**
	
	(20)	(26)	(27)	(1.94-2.45)	(1.45-1.98)

^1 ^Age adjusted mortality rates were estimated by the direct method, using the 2000 Campinas total population age structure as the standard.

^2 ^The number of deaths in each socioeconomic stratum is indicated in parenthesis.

^3 ^Significant differences at the 5% level are indicated in bold type.

## Discussion

The analysis of mortality rates in Campinas stratified by socioeconomic strata revealed that the highest mortality rates were concentrated in areas with poor living conditions. This study also identified the causes of death and age groups with large mortality differences between strata for each sex.

Consistent with previous research [1,3,4,13,18,24], profound social inequalities were evident for most causes of death, with a gradient of increasing mortality with decreasing socioeconomic status. Several other authors have also confirmed a distinct pattern of social inequalities in mortality by age [2,14,15], gender [7] and cause of death [11,15,25].

The social gap in mortality was greater among youth and adult populations than among the elderly population, although social differences in mortality have remained high among older age groups. In European countries, educational inequalities in mortality decreased consistently with increasing age, reducing rate ratios from 1.98 among males aged 30-39 years to 1.18 among males aged 80-89 years [14]. This pattern differs from a study conducted in New Zealand [2], where the social class mortality gradient among men aged 15-64 years was as strong in the older age groups as in the younger age groups. Some studies have demonstrated persistency and even an increase in social inequalities in mortality among the population aged 60 years or older. This suggests that unequal living conditions continue to influence health in later life, and that the patterns and levels of mortality among the elderly are the result of lifelong social inequalities in health [26].

Unlike the other causes of death investigated, female breast cancer mortality was higher in the high stratum than in the low stratum. These results confirm the findings of many studies [1,6,11,15,27] that observed decreasing breast cancer mortality rates from upper to lower socioeconomic levels. This may be related to different fertility patterns among high socioeconomic status women, such as delayed childbearing and nulliparity [27]. In a systematic review, Weir et al. [28] identified other relevant risk factors for breast cancer, such as heavy alcohol intake, postmenopausal obesity, high total caloric intake, hormone replacement therapy, and current use of oral contraceptives. In Brazil, delayed childbearing, current use of hormone replacement therapy, and greater frequency of alcohol intake are more prevalent among higher-educated women than lower-educated women [29-31].

Although a well-defined pattern of mortality according to socioeconomic strata was not established for prostate cancer, there was a remarkable gap between the low and high socioeconomic strata. Our findings reinforce those of a previous study [15]. However, an individual-based study conducted in Sweden showed increased mortality rates from prostate cancer among men with a high socioeconomic status [11]. Possibly, the high mortality rates among men living in poor areas of Campinas can be related to a lack of access to preventive cancer screening. Educational differences in prostate cancer screening practices were observed in the state of São Paulo [32,33]. Among men with 12 years or more of schooling, 56.8% reported undergoing a prior prostate-specific antigen (PSA) test or digital rectal examination, while this proportion was only 35.2% among low-educated men [33]. In addition to the highest prostate cancer mortality rates in the low stratum, this evidence of inequalities in screening practices is a strong argument for ensuring early detection and timely treatment of prostate cancer among men living in poor conditions.

The absence of a socioeconomic gradient in male lung cancer mortality in the city of Campinas differs from studies in developed countries that have reported increasing mortality rates with decreasing socioeconomic status [11,13,15,34]. According to Adler & Ostrove [8], in developed countries smoking was more prevalent in the upper social classes with subsequent spread across the other social strata. However, with the advent of intensive anti-smoking campaigns, a sharp drop in smoking rates occurred among more advantaged individuals, accompanied by a significant reduction in lung cancer mortality in this group. In Brazil, thanks to national tobacco control campaigns implemented in the early 1990s, a greater decline in smoking prevalence has been observed among those in higher socioeconomic groups than among those in lower socioeconomic groups in the last two decades [35]. As in many Brazilian cities, the smoking prevalence pattern has changed in Campinas, with a greater proportion of smokers in the lower-educated group than in the higher-educated group (59.3% *versus *23.0% respectively among men aged 20-49 years) [36]. Considering the long latency period of cancer, our study did not capture a social gradient in mortality, because not enough time has passed for social differences in mortality to be evident. However, it is quite possible that a cohort effect will soon appear and produce a social gradient in lung cancer mortality, with an increase in mortality rates in the lower-income groups. Thus, in addition to anti-tobacco campaigns focusing on the general population, there is a need for policies and interventions specifically designed for the most deprived groups, targeting improved access to health care and smoking cessation services, as well as ensuring adherence to anti-smoking treatments.

Cardiovascular mortality rates were 36% and 71% higher in low stratum areas than in wealthier areas for males and females respectively. Among both sexes, mortality rates from acute myocardial infarction and cerebrovascular diseases were higher in the low stratum than in the high stratum. These results are consistent with findings of previous studies [1,5,12,16], with variations in the magnitude of the difference. Among the adult population residing in selected Brazilian cities, Ishitani et al. [21] found an inverse association between education and cardiovascular mortality, mainly for cerebrovascular and hypertensive diseases. In the United States [16], cardiovascular mortality rates among men and women living in the most disadvantaged areas were 79% and 94% greater respectively in comparison with the least disadvantaged areas.

There are several explanations for the highest mortality rates in the low and middle strata than in the high stratum. The risk factors for cardiovascular diseases, such as physical inactivity and unhealthy diet, are more frequent among those with lower income and education [37]. Another explanation is the low rate of access to and use of health services by the population living in poverty, creating barriers to the implementation of prevention programs aimed at this group [15,33]. Because the prognoses of cerebrovascular diseases are closely related to health care, the lack of access to primary and emergency care, and poor quality of health services can widen social differences in mortality [1]. Therefore, the highest concentration of these avoidable deaths in the most-deprived areas highlights the need for more effective and specific interventions to ensure equity in prevention and control of cerebrovascular diseases.

Large socioeconomic differences were found for respiratory diseases, chronic obstructive pulmonary diseases, and pneumonia among both sexes. These marked social gradients, which are also reported in other studies [11,15,34], may be partly explained by smoking prevalence, genetic predisposition, and environmental and occupational exposures among socioeconomic groups. Our results also demonstrated that rate ratios for these diseases were higher among females than among males. This difference by sex is partly due to trends in the prevalence and intensity of smoking in Brazil. According to a study of trends in smoking indicators between 1989-2003, although a similar reduction in smoking prevalence has been observed among both sexes, those with higher-education reached a lower smoking prevalence than those with lower-education (18.1% *versus *32.2% respectively, among men and 13.4% *versus *22.8% respectively, among women) [35]. In addition, among the smokers, only low-educated women did not show a decline in the mean number of daily cigarettes consumed.

In relation to homicide among men, similar with Campinas, studies conducted in other big Brazilian cities have observed that mortality rates increase with deteriorating living conditions [19,20,38-40], indicating that violence is a constant threat, particularly among men. This explains the fact that in Campinas the male homicide rate was 2.44 times higher in the low stratum than in the high stratum.

Traffic accidents were 64% more frequent among males residing in the most-deprived than in the least-deprived areas. Previous Brazilian studies, however, have shown high mortality rates from traffic accidents in affluent areas [20,38]. The divergent data in Campinas can be partly explained by an increase in motorcyclist mortality since 2003 [41], which is related to the rapid growth of the motorcycle fleet. This growth is mainly driven by the low-income population, who have adopted the motorcycle because of its low cost and speed advantage.

Another important finding was the male excess mortality in all ages and groups of causes of death, indicating an accentuated sex inequality in mortality. Male premature mortality was also observed among young adults aged 20-29, whose mortality rates were around five times higher than the female rates in poor areas (2.16 *versus *0.42 deaths per 100 000 inhabitants). These results can be partly explained by male behaviour, which is shaped by social constructs of masculinity, defined in turn by social and cultural factors. Not only is there greater exposure to violence and lower rates of health services use among men than women, but they are also more likely to adopt unhealthy behaviours (e.g. alcohol abuse and driving under the influence of alcohol) [6,42].

However, although mortality rates were higher among males than among females, it is worth noting that rate ratios between low and high strata were larger in women than in men, with the exception of external causes. Among specific diseases, the sex contrast was striking for acute myocardial infarction, cerebrovascular diseases, pneumonia, and chronic lower respiratory diseases. For instance, for chronic lower respiratory diseases, the rate ratios between low and high strata were 1.31 (95% CI 1.15-1.48) among men and 2.19 (95% CI 1.94-2.45) among women, despite higher male mortality rates. Mackenbach et al. [6], in an individual-based study encompassing several countries, including the United States, Finland, and Italy, identified that educational differences in mortality were more accentuated among women than among men only for cardiovascular diseases, particularly for ischemic heart disease. In France, Rey et al. [24] found that socioeconomic mortality differences for external causes, respiratory diseases, and digestive diseases were higher among men than among women. Unlike other authors, our results demonstrated a wide social gap among women for most causes of death, despite their lower rates when compared with men.

We hypothesize that this phenomenon is the result of an interaction between gender roles and socioeconomic status. Female lifestyles are more conducive to good health, which explain low mortality rates in all socioeconomic strata. However, it is well known that poor people face barriers to access to health services, for example, no health insurance, difficulties paying medical bills, excessive waiting time in public clinics and hospitals, and long waiting lists for specialised care and diagnostic and therapeutic support services. Higher-income women can take advantage of private medical services. These factors, in addition to poor quality health care, may widen the social differences in mortality among women. On the other hand, gender-role socialization encourages males to adopt behaviours more injurious to health, which are disseminated among all socioeconomic groups. Consequently, although men experience higher mortality rates than women, the socioeconomic differences in male mortality are smaller.

Regarding methodological limitations of this study, it can be argued that by using the primary health care area as the unit of analysis, the results cannot be attributed to individuals. Since the mortality rates refer to the average of each stratum, the respective residents are not exposed to the same mortality risks. Considering the lack of updated demographic and socioeconomic data for the general population, the 2000 Census variables were used to define the socioeconomic strata. Thus, possible changes in the socioeconomic sphere may have occurred over time. Another limitation regarding the use of primary health care areas is that they may contain some internal heterogeneity, despite the strata's relatively homogeneous makeup.

However, the strength of this methodological approach is its adequacy for the demands of health administration for the assessment, surveillance, and implementation of policies and programs. In addition, it is worth noting the high quality of mortality data in Campinas, with a proportion of deaths assigned to ill-defined causes of less than 3%. Mortality estimates by strata were not underestimated, since only 1.4% of deaths occurring between 2004 and 2008 in Campinas could not be assigned to a specific health care unit area.

Based on our results, the main strategy used to narrow the social differences in mortality should be the reduction of socioeconomic inequalities, considering that living conditions have a direct influence on health [3,5]. According to Pearce et al. [2], these actions would promote lifestyle changes that decrease exposure to several risk factors, such as smoking and physical inactivity. Consequently, the reduction of socioeconomic inequalities would create a strong positive impact on reducing social disparities in mortality.

Another social determinant of health that partially explains the social inequality in mortality is the coverage and access to high-quality health care, including access to preventive and curative services, pharmacological therapies, and other technologies [1,8,33]. Therefore, another strategy to improve health outcomes for lower-income groups would be to increase the supply of health services and reduce possible differences in the quality of care [5].

## Conclusions

The socioeconomic disparities within the city of Campinas have led to an uneven distribution of mortality rates, with gaps of different magnitude according to sex, age, and cause of death. The identification of mortality differences among social groups is extremely useful to the health sector agenda, because municipal governments can respond appropriately to the specific needs of each region, and thus contribute to a reduction in social inequalities in mortality.

Nevertheless, interventions should not be restricted to the health field, but should also encompass other sectors of the public sphere. For instance, in the struggle against social inequalities in mortality from external causes, it is crucial that there be cooperation between public security organizations and public health agencies in the implementation of effective activities, by paying particular attention to the poor areas.

Evidently, reducing inequalities in living conditions should be the main goal to improve health conditions and to narrow social disparities in mortality. Social policies designed to reduce the social gap among population groups will have positive impacts on health conditions, and are an important strategy for the assurance of better health conditions among the general population.

In conclusion, given that social inequalities in mortality rates are an expression of the local socioeconomic situation, socioeconomic indicators can assist in planning programs designed to promote improvement in living conditions and to ensure access to quality health care, thus achieving equity in health conditions in the future.

## Competing interests

The authors declare that they have no competing interests.

## Authors' contributions

All authors have made substantial contributions to conception of this study and to analysis and interpretation of data. All authors read and approved the final manuscript.

## Pre-publication history

The pre-publication history for this paper can be accessed here:

http://www.biomedcentral.com/1471-2458/12/39/prepub
